# Measuring health expectancy in the European Union

**DOI:** 10.1093/eurpub/ckac129.067

**Published:** 2022-10-25

**Authors:** JV Santos, J Viana, B Devleesschauwer, JA Haagsma, C Costa Santos, W Ricciardi, A Freitas

**Affiliations:** 1 Public Health Unit, ACES Espinho/Gaia, ARS Norte, Porto, Portugal; 2 MEDCIDS, Faculty of Medicine, University of Porto, Porto, Portugal; 3 CINTESIS, Centre for Health Technology and Services Research, Porto, Portugal; 4 Department of Epidemiology and Public Health, Sciensano, Brussels, Belgium; 5 Department of Veterinary Public Health and Food Safety, Ghent University, Merelbeke, Belgium; 6 Erasmus MC, University Medical Center, Rotterdam, Netherlands; 7 Section of Hygiene, Institute of Public Health, Università Cattolica del Sacro Cuore, Rome, Italy

## Abstract

**Background:**

Healthy life expectancy (HLE) is a population health measure that combines mortality and morbidity, which can be calculated using different methods. In this study, we aimed to assess the correlation, reliability and (dis)agreement between two estimates monitored in the European Union (EU), that is, the European Commission's HLE based on self-perceived health (SPH-HLE) and the Institute for Health Metrics and Evaluation's HLE based on disability weight (DW-HLE), by sex, and comparing these results with LE and proportion of life spent in good health (%GH).

**Methods:**

We performed a retrospective study in the EU28 countries, between 2010 and 2017. The HLE methods differ in definition, measurement and valuation of health states. While SPH-HLE relies directly on one question, DW-HLE relies on epidemiological data adjusted for DW. Spearman's r, intraclass correlation coefficient, information-based measure of disagreement and Bland-Altman plots were used to assess reliability, correlation and disagreement in HLE resulting from both methods and in LE or %GH measured by both institutions.

**Results:**

Correlation and reliability between SPH-HLE and DW-HLE were good (better for males), with low disagreement, and were even better for LE between both institutions. The HLE Bland-Altman plots suggest a variability range of approximately 6 years for both sexes, higher for females. There was also an increasing HLE difference between methods with higher average HLE for both sexes.

**Conclusions:**

We showed wide variations between both methods with a clear and different high impact on female and male HLE, showing a tendency for countries with higher health expectancies to yield larger gaps between SPH-HLE and DW-HLE.

Acknowledgements: This presentation was supported by National Funds through FCT - Fundação para a Ciência e a Tecnologia,I.P., within CINTESIS, R&D Unit (reference UIDP/4255/2020)

**Key messages:**

• Different methods for evaluating health expectancy lead to significantly different results.

• There is a systematic tendency with countries with higher health expectancies to yield larger gaps between SPH-HLE and DW-HLE.

